# Clinical characteristics and genetic analysis of gene mutations in a Chinese pedigree with Peutz‐Jeghers syndrome

**DOI:** 10.1002/ccr3.2073

**Published:** 2019-03-03

**Authors:** Yudian Qiu, Tao Xuan, Mujun Yin, Zhidong Gao, Peng Guo, Xi Chen, Yingjiang Ye, Zhanlong Shen

**Affiliations:** ^1^ Peking University People's Hospital Beijing China; ^2^ Geneplus Co. Ltd Beijing China; ^3^ Department of Gastroenterological Surgery Peking University People's Hospital Beijing China

**Keywords:** germline variants, high‐throughput sequencing, Peutz‐Jeghers syndrome, somatic gene variants, STK11

## Abstract

The genome‐wide sequencing information of PJS is still lacking. Our result demonstrates that c.862+2T>C variant on STK11 as an important foundation of molecular mechanism in this familial PJS. Variants in KDR and MLL3 may play important roles in the initiation and development of this familial PJS polyps.

## 1 INTRODUCTION

Peutz‐Jeghers syndrome (PJS) is a rare autosomal dominantgenetic disease, the incidence of which has been estimated to be approximately 1 in 50 000‐200 000 births,^1^ it is characterized by the mucocutaneous melanin pigmentation, multiple gastrointestinal polyps, and elevated risk for cancer, involving benign and malignant tumors of multiple organs both in the gastrointestinal tract and extra‐gastrointestinal sites such as lungs, breasts, ovaries, and uterine cervixes. Recent studies suggest that the mutations of the gene STK11 located on the short arm of chromosome 19(19p13.3) are important molecular bases for the pathogenesis of PJS. Multiple germline mutations and somatic mutations have been identified in PJS patients.^2^ However, some studies show that in some patients the STK11 was not involved in PJS,^3^, ^4^, ^5^ which indicates that there are still other genes may be involved in this process. One recent study reported the existence of a heterozygous pathogenic variant of the DNA repair enzyme MUTYH in PJS patients,^6^ yet similar kind of research in this area is still lacking. In this research, we study the genetic information in the patient and her father's peripheral blood cells to screen for the existence of germline genetic mutations using high‐throughput sequencing technology and detect the somatic gene mutation in the patient's polyp specimens. Our study aims to find types of STK11 variant in our patient and detect any other types of variant. Our results could help this family avoid a high risk of having a child with PJS by preimplantation genetic testing, and would be helpful in predicting cancer risks and provide some meaningful guidance for the clinical work.

## 2 CASE PRESENTATION

**Figure 1:**
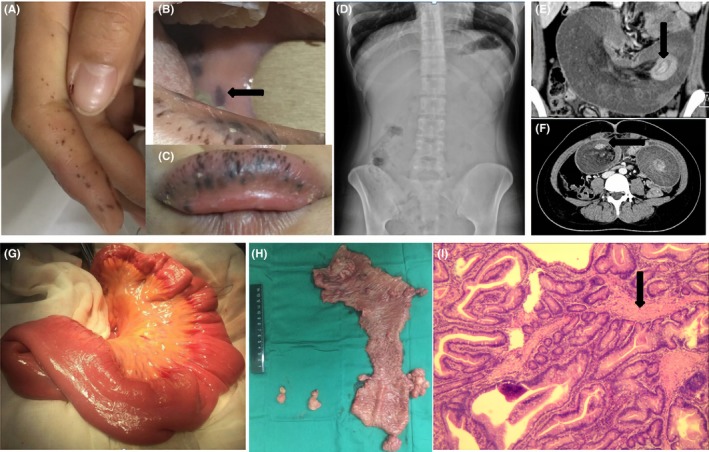
Pictures in patient's physical examination, the abdominal plain film, and contrast‐enhanced abdominal CT, pictures during the surgery and resected intestine, histopathological image of the patient. A‐C, Digits, oral mucocutaneous and lower lip pigmentation characteristic of PJS patient. D, Image from abdominal plain film demonstrating no significant expansion of intestinals, no organ injury or liquid gas plane. E, Image from contrast‐enhanced abdominal CT scan confirming small intestine‐to‐small intestine intussusception leaded by a polyp. F, Expansion and edema of the small intestine as well as multiple localized intraluminal polyp lesions existed. G, Image from transition part of edema intestine and normal intestine during the surgery. H, Image from surgical specimen from intestine resection with the lumen opened displaying the inner bowel mucosa and mass. I, Image from histopathological evaluation of the biopsy specimen obtained from the lesion suggests multiple regional adenomatous hyperplasia and interstitial focal lymphocyte infiltration, smooth muscle bundle can be seen around the gland

A 26‐year‐old young woman presented to our emergency department with chief complaint of abdominal pain with distension, vomiting with defecation stopped for 17 hours. She reported a history of intestine intussusception that had been cured 15 years ago. Pigmented macules over the lower lip, bilateral buccal mucosa, and digits with pale conjunctiva and hyponychiums were found on physical examination (Figure 1A,B,C). No significant expansion of intestines, no organ injury, or liquid gas plane was seen in the Abdominal plain film (Figure 1D), yet small intestine‐to‐small intestine intussusception led by a polyp was advised in the computed tomography (CT) (Figure 1E, arrows). The expansion and edema of the small intestine as well as multiple localized intraluminal polyp lesions were observed (Figure 1F, arrows). Microcytic anemia and hypoproteinemia were revealed by the laboratory examination, suggesting the disorders of digestion and absorption function as well as the chronic consumption state caused by the multiple PJS polyps. The diagnosis of PJS was established based on the European consensus statement.^7^ An emergency surgery (exploratory laparotomy) was performed to relieve intestinal obstruction. The patient returned to the intensive care unit after the surgery. Routine blood test, blood biochemical and arterial blood gas were monitored every day. An abdominal enhanced CT scan performed 3 days after the surgery showed the previous obstruction had been lifted. In addition, her father was diagnosed as PJS and underwent endoscopic polypectomy 5 years ago.

## 3 MATERIALS AND METHODS

Ten milliliter blood was taken from both the patient and her father after their approvals. The blood samples, together with the samples of biopsy, were subsequently tested with the high‐throughput sequencing, the somatic sequencing NGS results were derived from one polyp. The total DNAs of polyp tissues and peripheral blood cells were extracted according to standard protocols and were sheared to 300‐bp fragments with a Covaris S2 ultrasonicator. Indexed Illumina NGS libraries from tissues and the blood cells were prepared using KAPA Library Preparation Kit (Kapa Biosystems, Wilmington, MA, USA). Target enrichment was performed with a custom SeqCap EZ Library (Roche NimbleGen, Madison, WI, USA).

The capture probe used for detecting germline mutation was designed based on genomic regions of 58 genes, and the other probe used for detecting somatic mutation was designed based on genomic regions of 1021 genes most frequently mutated in colon tumor and other common solid tumors. The 58 genes and 1021 genes we studied is commercial set, part of the OncoH and OncoD product separately. Capture hybridization was carried out according to the manufacturer's protocol. Following hybrid selection, the captured DNA fragments were amplified and then pooled to generate several multiplex libraries.

Sequencing was carried out using Illumina 2 × 75 bp paired‐end reads on an Illumina HiSeq 2500 instrument according to the manufacturer's recommendations using TruSeq PE Cluster Generation Kit v3 and the TruSeq SBS Kit v3 (Illumina, San Diego, CA, USA).

After removing terminal adaptor sequences and low‐quality data, the reads were mapped to the reference human genome and aligned. GATK (https://www.broadinstitute.org/gatk/, The Genome Analysis Toolkit) and MuTect were employed to call somatic small insertions and deletions (indels) and single nucleotide variants (SNVs) by filtering blood cell sequencing data. Contra was used to detect copy number variations, and local algorithm was used to detect cancer‐associated structure variations.

## 4 RESULTS

### 4.1 Findings in the operation

The emergency surgery revealed an intussusception that was 2.6 m from the ileocecal valve, which was caused by an approximately 1 cm × 2 cm sized pedunculated polyp, the proximal intestines were dilated and edematous with multiple polyps of various sizes explored outside the intestinal duct. The highest position of the polyps reached 40 cm from the treze ligament. Five of the polyps were larger than 1cm, the largest reached 3cm×4cm. No ischemic necrosis was seen (Figure 1G). We performed partial resection of small intestine and intestinal anastomosis as well as the local resection to remove the adhesions and reduce the intussusception. A 30cm length of intestinal tube with three big polyps (≥1 cm) and dozens of small polyps (＜1 cm), as well as two separate large polyps, was resected. The specimen is shown (Figure 1H). The operation went smoothly, and the patient was sent to the intensive care unit for recovery after the surgery.

### 4.2 Histopathological results

Histopathological evaluation of the biopsy specimens obtained from the lesion suggested multiple regional adenomatous hyperplasia and interstitial focal lymphocyte infiltration. Smooth muscle bundle can be seen around the gland (Figure 1I), which corresponds to the typical characteristics of Peutz‐Jeghers type hamartomatous polyps.

### 4.3 Sequencing results

Among 58 types of germline genetic mutations we detected, only STK11 gene variant was detected in peripheral blood samples of the patient and her father. One single‐base substitution type of heterozygous variant (c.862+2T>C) was found in the patient and her father (Table 1). Somatic gene mutations of patient's polyps tissue were detected, using the DNA extracted from the patient's blood cells as the control. Only two somatic variants were found: KDR (c.1699G>A，p.V567M) and MLL3(c.4035G>T，p.K1345N). The frequencies of the two variants were 15.31% and 14.47%, respectively (Table 2).

**Table 1:** Germline genetic mutations of blood cells

	Start	End	Gene	cHGVS	pHGVS	Function	Frequency (%)	Homo/Heter
Patient	1 221 340	1 221 341	STK11	c.862+2T>C	‐	Splice‐5	49	Heter
Patient's father	1 221 340	1 221 341	STK11	c.862+2T>C	‐	Splice‐5	47	Heter

cHGVS, nucleic acid Human Genome Variation Society; Heter, heterogeneous; Homo, homogeneous; pHGVS, protein Human Genome Variation Society.

**Table 2:** Somatic gene mutation of patient's polyps

	Gene	cHGVS	pHGVS	Function	Maploc	Frequency (%)
Patient	KDR	c.1699G>A	p.V567M	Missense	4q12	15.311
Patient	MLL3	c.4035G>T	p.K1345N	Missense	7q36.1	14.471

cHGVS, nucleic acid Human Genome Variation Society; Maploc, Maplocation; pHGVS, protein Human Genome Variation Society.

## 5 DISCUSSION

Many literatures have reported that variants in STK11 are important molecular basis for the pathogenesis in PJS patients, more than 300 STK11 pathogenic variants have been reported according to the Human Gene Mutation Database. The STK11 gene variant we detected was located on site c.862+2T>C, it is a kind of missense mutation which was once reported in a PJS patient,^8^ and recorded in the ZJU‐CGGM database (http://www.genomed.org/lovd2/variants.php?select_db=STK11&action=view&view=0002938%2C0000453%2C0). No somatic mutations, copy number variation (CNV), or loss of heterozygosity (LOH) of STK11 were detected other than c.862+2T>C in our assays. No structural variation (SV) or copy number variation of other tested genes was detected either. We believe that site c.862+2T>C variant of STK11 gene is an important molecular basis for pathogenesis in the patient and her father.

KDR plays a key role in angiogenesis, and is closely related to the recurrence, metastasis and prognosis of a variety of tumors.^9^ The expression of VEGF/KDR is closely related to the metastasis of colorectal cancer.^10^ MLL3 catalyzes the monomethylation of large amounts of histone H3K4 and is known as a tumor suppressor gene. The mutations of MLL3 were found in a variety of tumors such as breast cancer, rectal cancer, retinoblastoma, gastric cancer, bladder cancer, and liver cancer.^11^, ^12^, ^13^, ^14^ The mutations of MLL3 in these tissues result in a declined methylation level of H3K4. This reduces the activation of the enhancer, resulting in the inactivation of some kinds of tumor suppressor genes, involving in tumorigenesis.^15^ Vogelstein et al^16^ revealed that colorectal cancers result from the sequential accumulation of mutations in oncogenes and tumor suppressor genes, mutations in at least four or five genes are required to produce a malignant tumor, the total accumulation of changes is responsible for determining the tumor's biologic properties. So the mutations in KDR and MLL3 are not enough to enable the patient progressed to the early stages of tumor development up to now.

PJScan cause chronic bleeding and anemia as well as recurrent obstruction and intussusception. Individuals with PJS are at increased risk for various kinds of tumors, especially epithelial neoplasias.^17^ A large number of the previous articles on PJS gene analysis only use techniques such as single‐gene testing and PCR, while genome‐wide sequencing information is still lacking. In addition, STK11 is complex and still being clarified, though the STK11 mutation is "pathogenic mutation," no clear genotype‐phenotype correlation has been demonstrated in PJS, studies of PJS families looking for other PJS locus suggested that in some the 19p13.3 locus was not involved in PJS.^4^, ^5^ This has raised the possibility of genetic heterogeneity. In this study, we performed a high‐throughput sequencing on the patient's and her father's peripheral blood cell samples to detect the germline mutations, and found the hereditary heterozygous variant of STK11 gene, site c.862+2T>C. The variants of somatic cells in polyp tissues were also detected, variant frequencies of KDR and MLL3 were 15.31% and 14.47%, respectively. We believe that site c.862+2T>C variant in the STK11 gene is an important foundation of molecular mechanism in this familial PJS. However, we did not detect any variants in the STK11 gene except c.862+2T>C in the patient's polyp tissues. The CNV and LOH of the STK11 gene were not detected either. At the same time, our detection of KDR and MLL3 gene variants in the patient's polyp tissues may also be involved in the formation and development of polyps, but the patient did not progress to the early stages of tumor development at present. Also, the clinical study has not yet found any signs of cancer in this PJS patient, but regular health management, prevention, and early screening of epithelial tumors especially colorectal cancer are recommended for the patient and her father. The results of this study could help this family avoid a high risk of having a child with PJS by preimplantation genetic testing.

## 6 CONCLUSION

Our result demonstrates that c.862+2T>C variant on STK11 as an important foundation of molecular mechanism in this familial PJS. Variants in KDR and MLL3 may play important roles in the initiation and development of the polyps, indicating a high risk of cancer. Our findings could help this family avoid a high risk of having a child with PJS by preimplantation genetic testing and could help clinicians better understand and provide better clinical surveillance and therapies for PJS patients.

## CONFLICT OF INTEREST

None declared.

## AUTHOR CONTRIBUTIONS

ZS and YQ: designed the report; ZS, YY, YQ, ZG and PG: performed the surgery; MY and XC: performed the pathological diagnosis; YQ and TX: analyzed the data and wrote the manuscript, All authors read and approved the final manuscript.

Consent for publication: The patient and her father included in this study allowed this paper to include some information of their disease for publication. Written informed consent for publication of the clinical details was obtained.
